# Total substitution and partial modification of the set of non-ribosomal peptide synthetases clusters lead to pyoverdine diversity in the *Pseudomonas fluorescens* complex

**DOI:** 10.3389/fmicb.2024.1421749

**Published:** 2024-08-19

**Authors:** Lucía Graña-Miraglia, Jorge Luis Geney Higuita, Juan Carlos Salazar, Diana Guaya Iñiguez, Carlos Alcolado León, Víctor A. García-Angulo

**Affiliations:** ^1^Department of Cell and Systems Biology, University of Toronto, Toronto, ON, Canada; ^2^Bacterial Metabolism Laboratory, Instituto de Ciencias Biomédicas, Microbiology and Mycology Program, University of Chile, Santiago, Chile; ^3^Laboratory of Enteropathogens, Instituto de Ciencias Biomédicas, Microbiology and Mycology Program, University of Chile, Santiago, Chile

**Keywords:** pyoverdine, non-ribosomal peptide synthetase, siderophore, ancestral character reconstruction, *Pseudomonas fluorescens*

## Abstract

Pyoverdines are high affinity siderophores produced by most *Pseudomonas* with a wide role in microbial interspecies interactions. They are primarily composed of a conserved chromophore moiety, an acyl side chain and a peptide backbone which may be highly variable among strains. Upon ferric iron sequestration, pyoverdines are internalized through specialized receptors. The peptide precursor of pyoverdine, termed ferribactin, is synthesized by a set of non-ribosomal peptide synthetase (NRPS) enzymes and further modified by tailoring enzymes. While PvdL, the NRPS responsible for the synthesis of the peptide moiety that derives into the chromophore is conserved, the NRPSs for the peptide backbone are different across fluorescent *Pseudomonas*. Although the variation of pyoverdine is a widely recognized characteristic within the genus, the evolutionary events associated with the diversity and distribution of this trait remain mostly unknown. This study analyzed the NRPSs clusters for the biosynthesis of the peptide backbone of ferribactin in the genomes of a representative subset of strains of the *Pseudomonas fluorescens* complex. Bioinformatic analysis of the specificity of adenylation domains of the NRPSs allowed the prediction of 30 different pyoverdine variants. Phylogenetic reconstruction and mapping of the NRPS clusters pinpointed two different general levels of modifications. In the first level, a complete replacement of the set of NRPRs by horizontal transfer occurs. In the second level, the original set of NRPSs is modified through different mechanisms, including partial substitution of the NRPS genes by horizontal transfer, adenylation domain specificity change or NRPS accessory domain gain/loss.

## 1 Introduction

In order to supply their needs for iron, many bacteria produce siderophores, specialized metabolites functioning to chelate ferric iron with high affinity to introduce it into the cell (Gomes et al., 2024). The main siderophores of the *Pseudomonas* genus are the pyoverdines. These are a family of fluorescent molecules that display extremely high affinity for Fe^3+^ (Cox and Adams, 1985; Schalk and Perraud, 2023). These siderophores are involved in several bacterial physiological traits such as virulence and biofilm formation (Yang et al., 2009; Lear et al., 2022; Chen et al., 2023; Jeong et al., 2023) and comprise important mediators of bacterial community assemblies (Figueiredo et al., 2022).

Bacteria synthesize and secrete pyoverdine upon iron deprivation. After chelation of Fe^3+^, ferripyoverdine is internalized into the periplasmic space by a dedicated TonB-dependent transporter composed of a ß-barrel outer membrane protein, termed FpvA in *Pseudomonas aeruginosa* (Poole et al., 1993; Meyer et al., 2002). In the periplasm, ferric iron is concomitantly reduced into Fe^2+^ and released to get translocated into the cytoplasm. Free pyoverdine is then exported through the PvdRT/OpmQ efflux complex to be recycled in iron uptake (Ringel and Brüser, 2018; Stein et al., 2023).

To date, around 100 different pyoverdine structures are known (Ghssein and Ezzeddine, 2022). These molecules are composed of three structural features: a hydroxyquinoline chromophore core, an acyl side chain and a peptide backbone. The chromophore is responsible for the fluorescence and is highly conserved between pyoverdine variants. The side chain is attached to the chromophore and may be composed of one of a few acyl chain types, such as malic or succinic acid. The peptide backbone represents the main source of structural divergence among pyoverdine forms as it may be very variable in sequence and length (Molina et al., 2016; Schalk et al., 2020; Ghssein and Ezzeddine, 2022). Pyoverdines with peptide backbones from 6 to 14 amino acids are known (Demange et al., 1990; Meyer et al., 2008; Ye et al., 2013; Rehm et al., 2022, 2023) and the production of pyoverdines with peptide chains as short as four amino acids has been predicted (Molina et al., 2016). The peptide backbone may include non-proteinogenic amino acids, with some variants displaying partial cyclization in the chain (Demange et al., 1990; Budzikiewicz, 2004; Ghssein and Ezzeddine, 2022). Pyoverdines coordinate iron through a catechol moiety conserved in the chromophore and also by hydroxamate and/or hydroxycarboxylate functional groups present in the peptide backbone-derived structure (Boukhalfa et al., 2006; Andrejević et al., 2023).

The core enzymes for pyoverdine synthesis are Type I non ribosomal peptide synthetases (NRPSs). Type I NRPSs are large, multimodule enzymes able to catalyze the formation of small peptides usually involved in specialized metabolism. In NRPSs, each module incorporates one amino acid into the resulting peptide. Within the module, an adenylation (A) domain selects and activates the amino acid substrate. Both proteinogenic and non proteinogenic amino acids can be substrates for A domains. Following the A domain there is usually a peptidyl carrier protein domain, also called thiolation (T) domain, which shuttles substrates between domains and modules. The basic structure of an NRPS module is completed by a condensation (C) domain, which catalyzes the peptide bond formation between the donor and acceptor substrates of its neighboring T domains (Brown et al., 2018). NRPSs modules may also contain additional tailoring domains, which could modify the non ribosomal peptide *in situ*. One of such domains is the epimerization (E) domain, which performs epimerization modifications in L-amino acids loaded into the enzyme to render D-amino acids-containing peptides (Miller and Gulick, 2016; McErlean et al., 2019). Normally, the non ribosomal peptide sequence is determined by the order of the modules in the NRPS, which is known as the colinearity rule (Wenski et al., 2022; Cesa-Luna et al., 2023). Also, the general specificity of A domains toward their amino acid substrate can be inferred with high accuracy by analysis of the sequence and structural features of experimentally characterized domains (Bachmann and Ravel, 2009). In many instances, the synthesis of a single non ribosomal peptide may require multiple NRPSs encoded in an operon. In such cases, all the enzymes involved are exquisitely coupled in a single assembly line (Stachelhaus and Marahiel, 1995; Hahn and Stachelhaus, 2004; Duban et al., 2022). These general principles have been widely applied to infer peptides resulting from NRPSs pathways (Cesa-Luna et al., 2023; Jian et al., 2023).

During pyoverdine biosynthesis, the peptide resulting from the core synthesis performed by the NRPSs is called ferribactin and is further modified in the periplasm by additional tailoring enzymes of the biosynthetic pathway to render the mature pyoverdine (Schalk et al., 2020; Sugue et al., 2022). The genes for the NRPSs and accessory and maturation enzymes, together with genes required for pyoverdine translocation may be clustered in a single locus or grouped in a few genetic clusters across the genome (Moon et al., 2008). The pyoverdine biosynthetic clusters contain different sets of NRPSs genes distinguishable by the structural segment they contribute to the ferribactin sequence. On one hand, the *pvdL* gene codes for a conserved NRPS responsible for the chromophore precursor sequence that initiates the ferribactin synthesis by adding the first three amino acids to the peptide backbone. This leader sequence is L-Glutamate/D-Tyrosine/D-Diaminobutyrate (L-Glu/D-Tyr/L-Dab) and is the same for the ferribactin of all pyoverdine variants (Mossialos et al., 2002; Schalk et al., 2020). The chromophore moiety is later derived from tailoring enzymatic modifications of the D-Tyr/L-Dab residues, while the acyl side chain is generated from alternative modifications of the initial L-Glu residue (Dorrestein et al., 2003; Budzikiewicz, 2004). On the other hand, a set of NRPSs encoded in a different part of the pyoverdine cluster or in a cluster different from the one where *pvdL* locates, adds the rest of the amino acids to the peptide chain. The number and module composition of these NRPSs is highly variable among different *Pseudomonas* species and ultimately determine the particular identity of each pyoverdine variant (Ringel and Brüser, 2018; Schalk et al., 2020; Dell'Anno et al., 2022). Usually, the cognate pyoverdine receptor *fpvA* gene is located downstream of the genes for these NRPSs. This receptor functions to recognize the native pyoverdine variant synthesized by the genes of the cluster, but may also bind and internalize other structurally-related xeno-pyoverdines (i.e. pyoverdines produced by other strains) (Greenwald et al., 2009; Schalk and Guillon, 2013; Schalk et al., 2020). Furthermore, the same strain could encode additional receptors in different loci able to uptake other pyoverdine forms and even different siderophores (Hartney et al., 2013; Chan and Burrows, 2023).

The ability to produce a high variety of pyoverdine forms is a striking attribute of the *Pseudomonas* genus. Accordingly, the pyoverdine biosynthetic locus was early identified as one of the most divergent loci in *Pseudomonas aeruginosa* strains genomes (Spencer et al., 2003). Later, it was shown that the *fpvA* gene is highly divergent and displays features of positive selection (Smith et al., 2005). The fact that pyoverdine comprises a public good, with competitor bacteria in the microbial community able to use it may be one of the reasons for its extreme variability. Cheater strains, which express the receptor to exploit the siderophore but lose the ability to biosynthesize it, have been shown to co-occur with producers in natural communities in soil and ponds (Butaite et al., 2017; Stilwell et al., 2018; Butaite et al., 2021). This has been postulated as one of the possible selection pressures for pyoverdine variation (Butaite et al., 2017).

Pyoverdine peptide backbone variants are thought to be species-specific with some species even having the potential to produce different variants (Meyer et al., 2008; Schalk and Guillon, 2013; Butaite et al., 2021; Rehm et al., 2022). *P. aeruginosa* strains have been documented to collectively produce four different pyoverdine types defined on the basis of the primary sequence of their peptide backbone (Schalk and Perraud, 2023). Consistently, the pyoverdine biosynthetic loci for the first three pyoverdine types (PVDI, PVDII and PVDIII) display peptide backbone NRPS genes with distinctive modular arrays. PVDIV likely derived from the PVDIII cluster through the loss of a module in one of the NRPSs (Smith et al., 2005). In a different example, at least five different NRPSs cluster arrangements for peptide backbone are found in *Pseudomonas putida* genomes. Such arrangements are proposed to be specific to *P. putida* as neither of them is present in the closest relative species (Molina et al., 2016). Owed to the high variety and specificity of pyoverdine production, the elucidation of pyoverdine identity has been proposed as a tool for taxonomic classification of *Pseudomonas* species, subspecies and strains and this process is called siderotyping (Meyer et al., 2002, 2008; Meyer, 2007; Mulet et al., 2008; Ye et al., 2013). Nonetheless, systematic studies to assess the richness and distribution of pyoverdine variants between species and the evolutionary paths leading to their high variation have only recently started.

Pyoverdine variation plays a crucial role in *Pseudomonas* niche adaptation and intermicrobial interactions. The arrangements of NRPS for the biosynthesis of this siderophore are complex and their evolutionary patterns are not easily recognizable in the genomes of the species. Understanding the mechanisms driving this diversity will provide essential insights into the evolution and adaptation of this ubiquitous genus. Furthermore, NRPSs variation is a general source of diversity for specialized metabolites with biotechnological potential (Bozhüyük et al., 2024). Therefore, knowledge of NRPS evolution is necessary to develop guidelines for designing novel bioactive metabolites.

*Pseudomonas* is one of the most populated bacterial genera, with more than 220 species with validly published names, including bacteria from a myriad of niches and with high metabolic diversity. The number of species included in this genus is constantly increasing and their inferred phylogenetic relationships are complex and under constant reassessment (Lalucat et al., 2020; Saati-Santamaría et al., 2021). *Pseudomonas fluorescens* composes one of the three main lineages within the *Pseudomonas* genus including more than fifty different assigned species (Garrido-Sanz et al., 2016, 2017; Lalucat et al., 2020). Many of the strains belonging to this lineage possess wide biotechnological potential as producers of bioactive molecules and as plant growth-promoting bacteria (Garrido-Sanz et al., 2017). The phylogeny of the strains of the *P. fluorescens* lineage resembles a species complex (Silby et al., 2009; Garrido-Sanz et al., 2016). Accordingly, the *P. fluorescens* complex has been suggested to be composed of nine different groups namely *Pseudomonas jessenii, Pseudomonas mandelii, Pseudomonas koreensis, Pseudomonas corrugata, Pseudomonas protegens, Pseudomonas chlororaphis, Pseudomonas gessardii, Pseudomonas fluorescens* and *Pseudomonas fragi* (Mulet et al., 2012; Garrido-Sanz et al., 2016). This study aims to leverage the close genetic relatedness of *P. fluorescens* to identify recent events leading to pyoverdine variation. This was achieved by performing a systematic assessment of the presence and modular composition of NRPSs in a set of strains belonging to this complex, followed by an assessment of the evolutionary mechanisms using phylogeny-based approaches.

## 2 Materials and methods

### 2.1 Genomic data

Seventy four representative species were selected from a whole-genome phylogeny based on nucleotide data from 93 sequenced strains belonging to the *P. fluorescens* complex (Garrido-Sanz et al., 2016). We chose one or more genomes from each clade within the eight phylogenomic groups defined using digital DNA-DNA hybridization (dDDH) and the clustering program OPTSIL in Garrido-Sanz et al. (2016). FASTA and annotation files for the genomes of these strains were retrieved from the Bacterial and viral bioinformatics resource center (BV-BRC; https://www.bv-brc.org/) (Olson et al., 2023).

### 2.2 Pyoverdine identification

The genomes of the *Pseudomonas* strains were screened for the NRPS genes for pyoverdine biosynthesis and pyoverdine receptors in the BV-BRC. The cognate pyoverdine receptor genes located downstream of the NRPSs genes were also retrieved. The modular structure of each NRPS was determined using the PKS/NRPS predictor (Bachmann and Ravel, 2009). The specificity of A domains was estimated by a consensus prediction using the NRPSpredictor2 and PKS/NRPS software (Bachmann and Ravel, 2009; Röttig et al., 2011) and a phylogeny-based prediction methodology reported before by grouping with A domains with experimentally determined substrates (Khayatt et al., 2020). The A domains with substrates that could not be predicted by this approach were designated Xxx.

### 2.3 Phylogenetic analysis

We used PIRATE (Bayliss et al., 2019) to create a pangenome and a core genome alignment of the 74 *P. fluorescens* complex strains. We run PIRATE on 60, 70, 80, 90, and 95 amino acid % identity thresholds. We identified the genes present in only one copy in all genomes. We then extracted and concatenated the nucleotide sequence alignments of the previously identified one copy orthologous genes. The resulting core genome alignment composed of 2,685 concatenated gene families was used to build a species tree using RAxML (Stamatakis, 2014) with parameters -f a -m GTRGAMMA -p 12345 -x 12345 -# 100.

The receptor amino acid sequences were aligned with FSA (Bradley et al., 2009) and a tree was built using RAxML with parameters -m PROTGAMMAGTR -p 12345 -# 20. A domains amino acid sequences were obtained using the information of the modular composition of the NRPSs obtained by PKS/NRPS and aligned with FSA and a tree was built using RAxML with parameters -f a -m PROTCATWAG -p 12345 -x 12345 -# 100. Visualization was performed in R using Ggtree (Xu et al., 2022). After the initial determination of specificity performed by the consensus prediction, the phylogenetic analysis indicated that the Gly domain group was polyphyletic, with one of the clades containing mixed Gly and Ala domains. In a second phylogeny of A domains including the domains of experimentally characterized pyoverdines, all the experimentally Ala domains mapped to this clade. Hence, the components of this clade were assigned to Ala specificity.

### 2.4 Ancestral character state reconstruction

Ancestral character state reconstruction was performed using the functions fitMk and fitgammaMk from phytools in R (Revell, 2024). fitMk, implements a Mk model (Markov model of trait evolution) in a maximum likelihood framework. The Mk model is a direct analog of the Jukes-Cantor model for sequence evolution. The model applies to a discrete character having k unordered states and for the basic Mk model, the instantaneous rates between all pairs of characters are equal. In fitgammaMk, the rates are assumed to have been sampled randomly from a discretized Γ distribution. We run three models, fitMK choosing the equal rates model (model = “ER”); fitMk choosing the symmetric model (model = “SYM”), which assumes that the rate of change from state A to state B is the same as the rate of change from state B to state A but it does not assumes that all pairs of states have the same rate; and fitgammaMk choosing a symmetric model where the rates of character evolution are allowed to vary according to a gamma distribution with 10 rate categories (model = “SYM,” nrates = 10). The models were compared using ANOVA in R and the selection was based on Akaike information criterion (AIC) values.

## 3 Results

### 3.1 Diversity of pyoverdine types produced by species of the *P. fluorescens* complex

We analyzed pyoverdine diversity and evolution within the *P. fluorescens* complex. Bacterial complexes refer to a group of strains that share genetic and evolutionary relationships but may not fit neatly into a single, distinct species category. These complexes are dynamic and can evolve through various mechanisms, including horizontal gene transfer, mutation and recombination. As a result, they can display a continuum of genetic variation rather than a clear separation into discrete species (Almeida and Araujo, 2013). They typically include closely related strains that show variations in their genetic makeup, traits and ecological adaptations. These characteristics allow us to assess the recent evolution of the rapidly evolving pyoverdine structure and identify processes involved in generating diversity.

**Figure 1:**
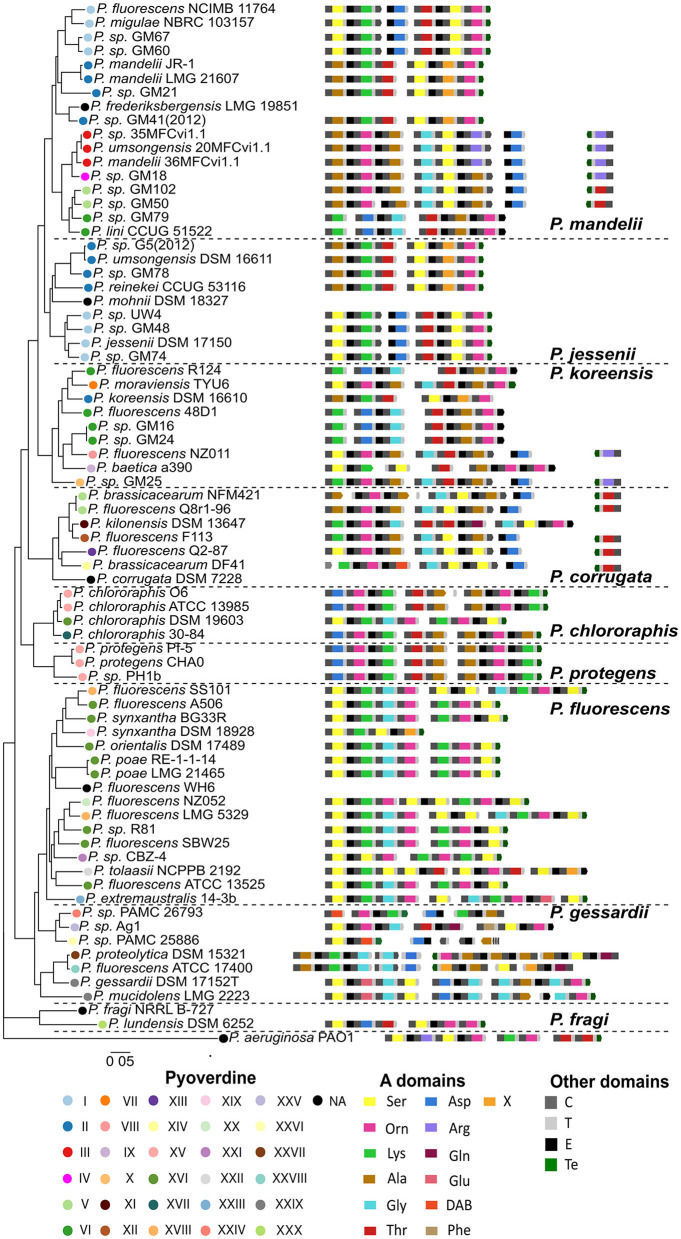
Phylogenetic tree of the set of the 75 *Pseudomonas* species based on the alignment of concatenated core gene families. The set of NRPSs for the ferribactin peptide backbone and their predicted modular composition is shown in front of each species. The color of the circles indicates the pyoverdine type. The colors of the A domains in NRPSs indicate their specificity. Other domains of the NRPSs are also indicated. Previously defined phylogenetic groups within the complex are indicated by dashed lines.

We selected 74 genomes that represent the *P. fluorescens* complex diversity plus the genome of *P. aeruginosa* PAO1 from the Bacterial and Viral Bioinformatics Resource Center (BV-BRC) (Olson et al., 2023). A pangenome analysis was performed and 2,685 orthologous single copy genes were identified. These genes were concatenated to obtain a core genome alignment with which we performed a phylogenetic reconstruction via maximum likelihood. The phylogenetic relationships among the representative subset of species allowed the recognition of the nine groups previously described to compose this complex (Figure 1) (Garrido-Sanz et al., 2016). The *P. mandelii, P. jessenii, P. koreensis, P. corrugata, P. protegens, P. chlororaphis, P. gessardii, P. fluorescens* and *P. fragi* clades are defined by the presence of type strains. Overall, the tree topology is similar to others reported for this complex (Mulet et al., 2012; Garrido-Sanz et al., 2016, 2017), except for the association of a subset of strains reportedly belonging to the *P. gessardi* group with the *P. fluorescens* group.

To get insights into the diversity of pyoverdines produced by the members of this complex, the sequences of the NRPSs for the peptide backbone in the pyoverdine biosynthetic cluster of each species in the databases were searched and retrieved. Each of the amino acid sequences was subjected to NRPS domain composition prediction using PKS/NRPS Analysis and NRPSpredictor (Bachmann and Ravel, 2009; Röttig et al., 2011). Next, the specificity of the A domains was predicted using the aforementioned software and a previously developed phylogenetic approach (Khayatt et al., 2020). The predicted primary peptide backbones of ferribactin for every strain are detailed in Figure 1 and Supplementary Table S1. Five strains were found to lack NRPS pyoverdine biosynthesis genes and were considered non-producers (indicated by black circles in Figure 1). The pyoverdines of eight strains included in this analysis have undergone experimental sequence determination (Demange et al., 1990; Wong-Lun-Sang et al., 1996; Moon et al., 2008; Hartney et al., 2013; Matthijs et al., 2016). Hence, a comparison was conducted between the primary sequence predicted for these pyoverdines and the reported ones (Supplementary Table S2). In this subset of pyoverdines, the nature of 59 out of 62 (95.1%) amino acid residues was accurately predicted by our approach.

As expected, a high diversity of pyoverdine backbones were predicted in this group of *P. fluorescens* representatives. This analysis identified 30 distinct pyoverdine variants based on the primary amino acid composition determined through the specificity of the A domains found in the pyoverdine loci. We detected 12 A domains with distinctive determined specificity, different combinations of these domains are responsible for the pyoverdine diversity. The length of the predicted peptide backbones varied from 4 to 12 amino acids in this group of strains. Notably, the variants encountered are not species-specific, and when mapped to the species tree, the distribution pattern of these pyoverdine types does not align with the phylogenetic distribution of species. Even though closely related strains are more likely to share the pyoverdine structure, the same structure can be found in different clades, and closely related strains can have different pyoverdine structure. Nonetheless, the topology of the phylogeny based on PvdL amino acid sequences, which synthesizes the invariant segment of the ferribactin, was congruent with the species tree topology (Supplementary Figure S1). This confirms that, unlike the downstream NRPSs responsible for building the peptide backbone, the enzyme PvdL stands out as the only conserved NRPS involved in synthesizing the chromophore precursor.

**Figure 2:**
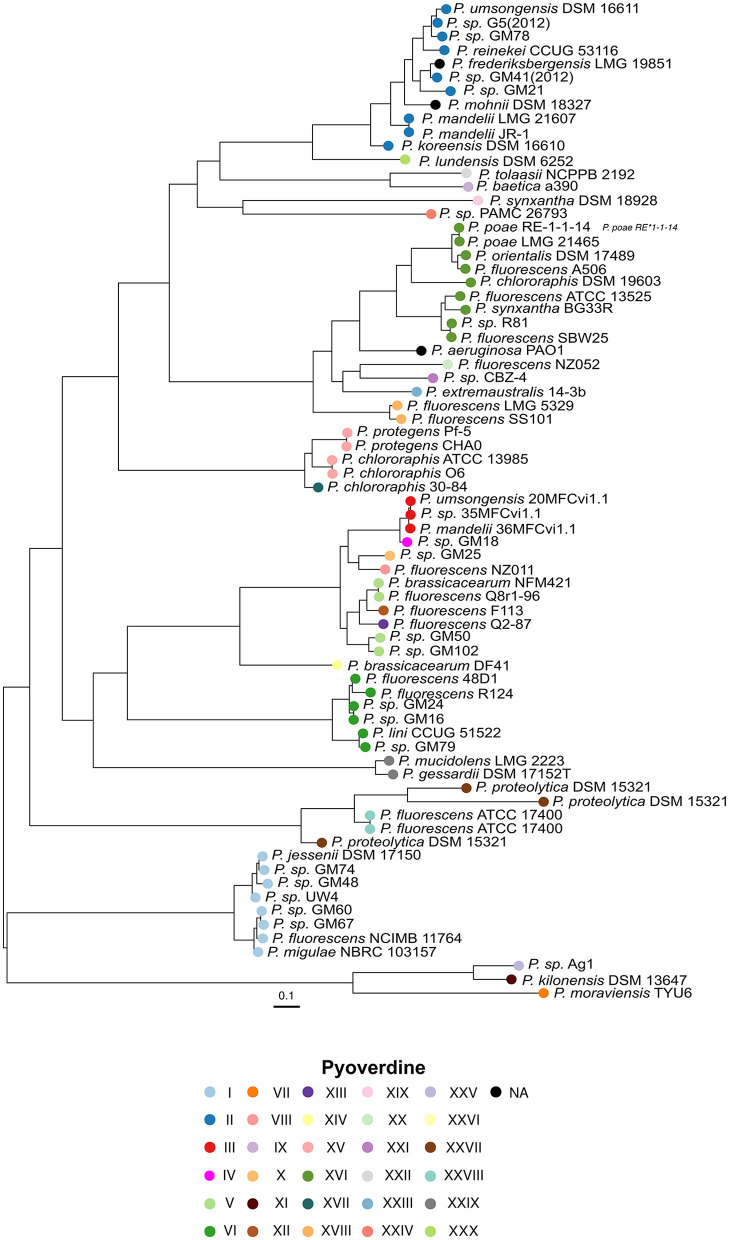
Unrooted phylogenetic tree based on the alignment of 72 pyoverdine receptor amino acid sequences. The circle colors indicate the pyoverdine synthesized by the corresponding strain as in Figure 1.

In addition, a phylogenetic tree was inferred from the amino acid sequences of the pyoverdine receptor encoded in the vicinity of the variable NRPSs genes, which is expected to recognize the native pyoverdine. Remarkably, this tree mainly groups receptors according to the type of variable peptide chain predicted and independently of the phylogeny of the species (Figure 2). The clades formed are composed of receptors that recognize structurally similar pyoverdines. This provides support for the assumption that these receptors function to recognize the endogenously synthesized pyoverdine and are also in agreement with the notion that differences in the peptide backbone are the main factor for pyoverdine structure diversity. Two out of the five pyoverdine non-producers, *P. frederiksbergensis* LMG 19851 and *P. mohnii* DSM 1832, still carry a pyoverdine receptor sequence. Strikingly, this analysis identified two strains with more than one receptor gene downstream the NRPSs genes. *P. proteolytica* DSM 15321 has three receptors and *P. fluorescens* ATCC 17400 has two. These two strains are closest relatives and their receptors cluster together in the tree: nonetheless, the three DSM 15321 receptors display a high phylogenetic distance from each other, while the two ATCC 17400 are closely related to each other and form a subcluster. This observation suggests the occurrence of gene duplication events, followed by rapid divergence in the case of DSM 15321.

We employed ancestral state reconstruction to infer the evolutionary trajectories of pyoverdines across the *P. fluorescens* group. Ancestral state reconstruction allows us to infer the traits of common ancestors, to understand the direction and rate of evolutionary changes, and to identify key transitions in the evolutionary history of a group. Such analyses can reveal patterns of trait evolution, provide insights into the adaptive significance of traits and help in understanding the evolutionary processes that drive diversity within lineages. By reconstructing ancestral states, we can gain a deeper understanding of the historical context of trait evolution and make more informed predictions about the evolutionary dynamics of related species.

**Figure 3:**
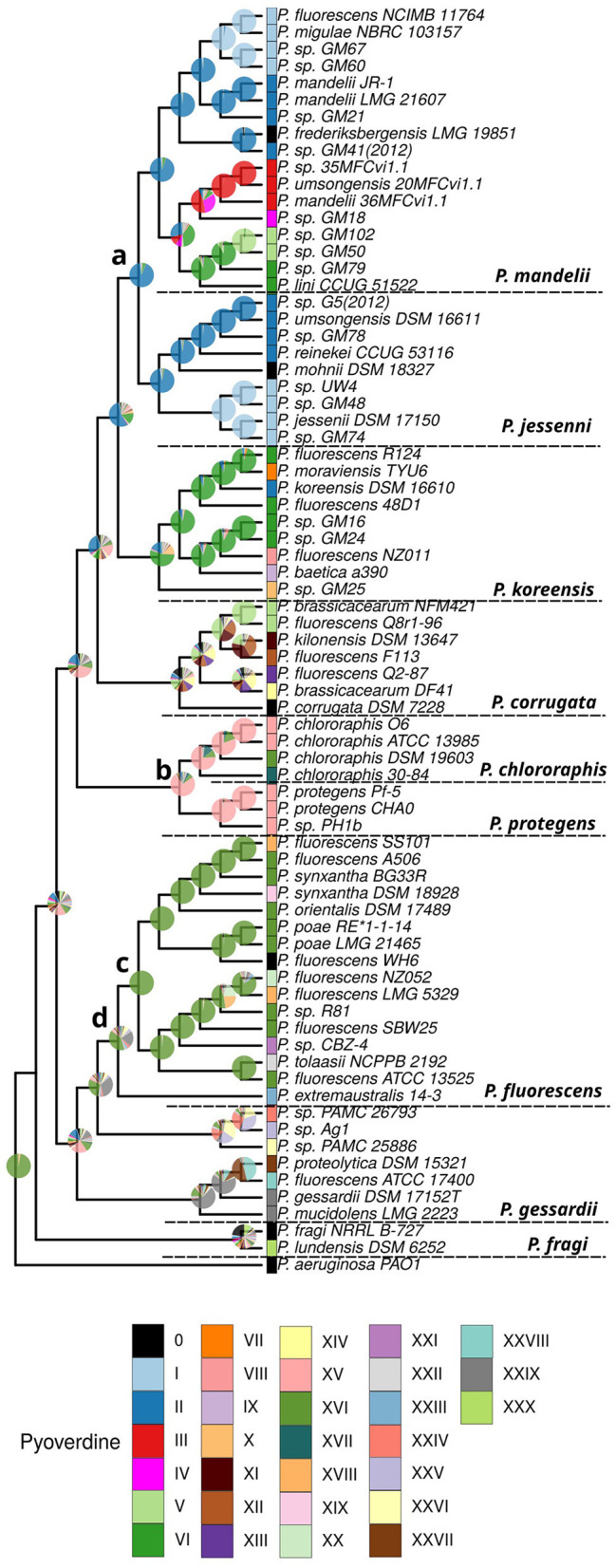
Reconstruction of the pyoverdine ancestral state in the *Pseudomonas fluorescens* complex. The inferred phylogeny of the species and the associated data on the pyoverdine types were used to perform an analysis of ancestral character state reconstruction. The pie charts illustrate the probabilities of ancestral pyoverdine types for each node. The relevant nodes and their most likely state are indicated by letters as follows: (a) ancestral character for the *Pseudomonas mandelii* and *Pseudomonas jessenii* groups, type II pyoverdine with 91% probability; (b) ancestral state for the *Pseudomonas chlororaphis* and *Pseudomonas protegens* groups, type XV, 74%; (c) ancestral state for the *P. fluorescens* strains bearing type XVI pyoverdine, type XVI, 99%; (d) most likely ancestral state for the whole *P. fluorescen*s group, type XVI, 38%. The results from an extended M*k* discrete character evolution model, with variable rates among edges of the tree taken from a discretized gamma distribution are shown.

We tested three different models (see Section 2) and selected the simplest one, the Mk model (Lewis, 2001) with equal rates, based on AIC values due to the high congruence of results among the models. Using the Mk model, we assessed changes in pyoverdines states on a phylogenetically informed framework, allowing us to trace the lineage-specific evolutionary pathways and to estimate trait states in ancestral species. Our analysis revealed that pyoverdine type II is likely the ancestral form for the P. *mandelii* and P. *jessenii* groups, with 91% probability (Figure 3, node a). In contrast, pyoverdine type XV emerged as the most probable ancestral state for the *P*. *chlororaphis* and *P. protegenes* groups with 74% probability (Figure 3, node b). Also, this analysis indicates that type XVI state is the common ancestor for all the *P. fluorescens* strains that bear it with a high probability (99%, Figure 3 node c). Moreover, type XVI was estimated as the most likely state for the whole *P. fluorescens* group (38%, Figure 3 node d). These findings provide robust support and context for the evolutionary hypotheses concerning the emergence and diversification of pyoverdine types across the *P. fluorescens* lineage (Figure 3).

Next, the phylogenetic tree of the species and its cognate data on the structure of NRPSs clusters were analyzed in order to identify putative changes leading to ferribactin primary sequence variations. We examined the NRPSs of closely related strains with variations in modules that lead to new pyoverdines being synthesized to identify the genetic mechanisms responsible for diversity. In general, two levels of modifications rendering the variation of pyoverdines were detected: the full substitution of the original set of NRPSs for the variable peptide backbone and the partial modification of the ancestral set.

### 3.2 Identification of evolutionary events leading to pyoverdine diversity by complete substitution of the NRPSs set

**Figure 4:**
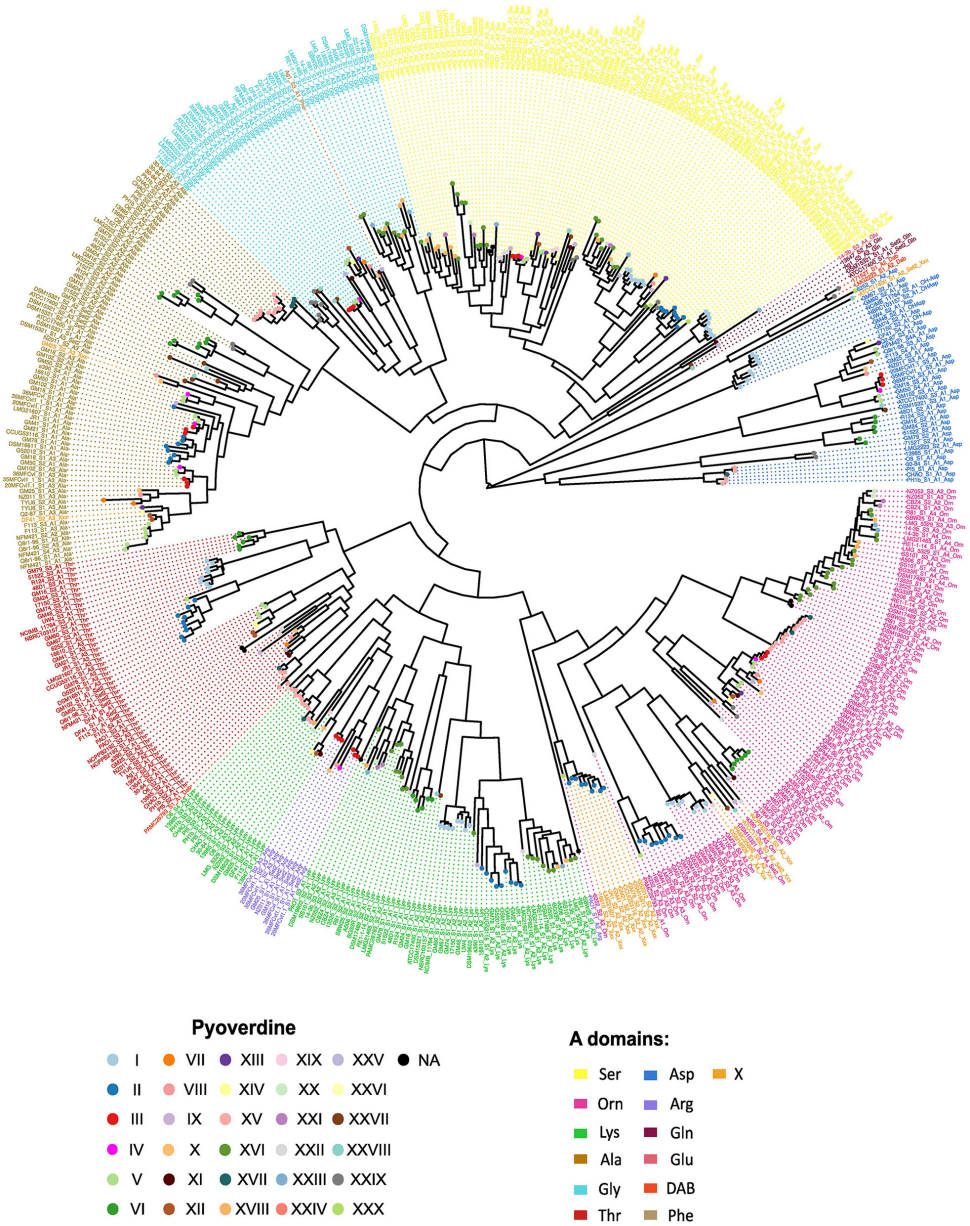
Unrooted phylogenetic tree based on the alignment of the amino acid sequences of 506 A domains. The phylogeny of A domains belonging to the NRPSs of the *Pseudomonas fluorescens* strains was inferred using RAxML. Font color indicates the predicted specificity and the pyoverdine type produced by the strain to which each domain belongs is indicated by circles. The nomenclature to term A domains followed is: *strain*_S*(NRPS number to which the domain belongs)*_A*(domain position in the NRPS)*_*Amino acid*. A high resolution image is provided in the electronic version of this article. The relevant subtrees for the analysis are magnified and shown in successive figures.

Given that pyoverdine structure is highly variable, it is usually assumed that its peptide backbones are species-specific. Nonetheless, according to the results of the predictions of A domains specificities, strains assigned to different species may produce the same ferribactin peptide backbone moiety. The most dramatic cases are those where pyoverdines of bacterial species belonging to different *P. fluorescens* phylogenetic groups have the same peptide chain. Six of such cases are detected. These correspond to pyoverdine type I (distributed in species of the *P. mandelii* and *P. jessenii* groups), type II (*P. mandelii, P. jessennii* and *P. koreensis*), type IV (*P. mandelii* and *P. corrugata*), type V (*P. mandelii* and *P. koreensis*), type XV (*P. protegens* and *P. chlororaphis*) and type XVI (*P. chlororaphis* and *P. fluorescens*; Figure 1). Independently of the position of the strains in the phylogeny of the species, the receptors associated with these pyoverdines grouped according to the type of peptide chain predicted (Figure 2), indicating that species divergent in the phylogenetic tree might produce analogous pyoverdines. The presence of the same pyoverdine type in different groups could be indicative that such type is ancestral and is vertically inherited and conserved by strains of the groups. Alternatives are that the pyoverdine NRPSs gene cluster was horizontally transferred or independently evolved twice. Notably, according to the ancestral character reconstruction analysis results, only the types II and XV seem to comprise pyoverdines which are ancestral in their groups and their presence in strains of two different groups (*P. protegens* and *P. chlororaphis* for type XV and *P. jessenii* and *P. mandelii* for type I) may be parsimoniously explained by vertical inheritance (Figures 1, 3 nodes a and b). In every other instance, the modules responsible for the pyoverdine peptide backbone could have been either horizontally transferred or independently evolved to generate comparable chains in species across distinct groups. To gain insights into these two possibilities for each of these types, a phylogenetic analysis of A domains was performed. The amino acid sequence of each A domain from all the NRPSs in the species of this study was retrieved and used to reconstruct a phylogenetic tree (Figure 4). In this tree, the A domains are clustered mainly according to their specificity. There are five major monophyletic clades corresponding to sequences loading amino acids Ser, Gly, Ala, Thr and Orn. The A domains for Arg form a subclade within the Lys clade. In subsequent analyses, relevant subtrees of this phylogenetic tree of A domains are amplified and shown in the figures to investigate the evolutionary hypothesis presented.

**Figure 5:**
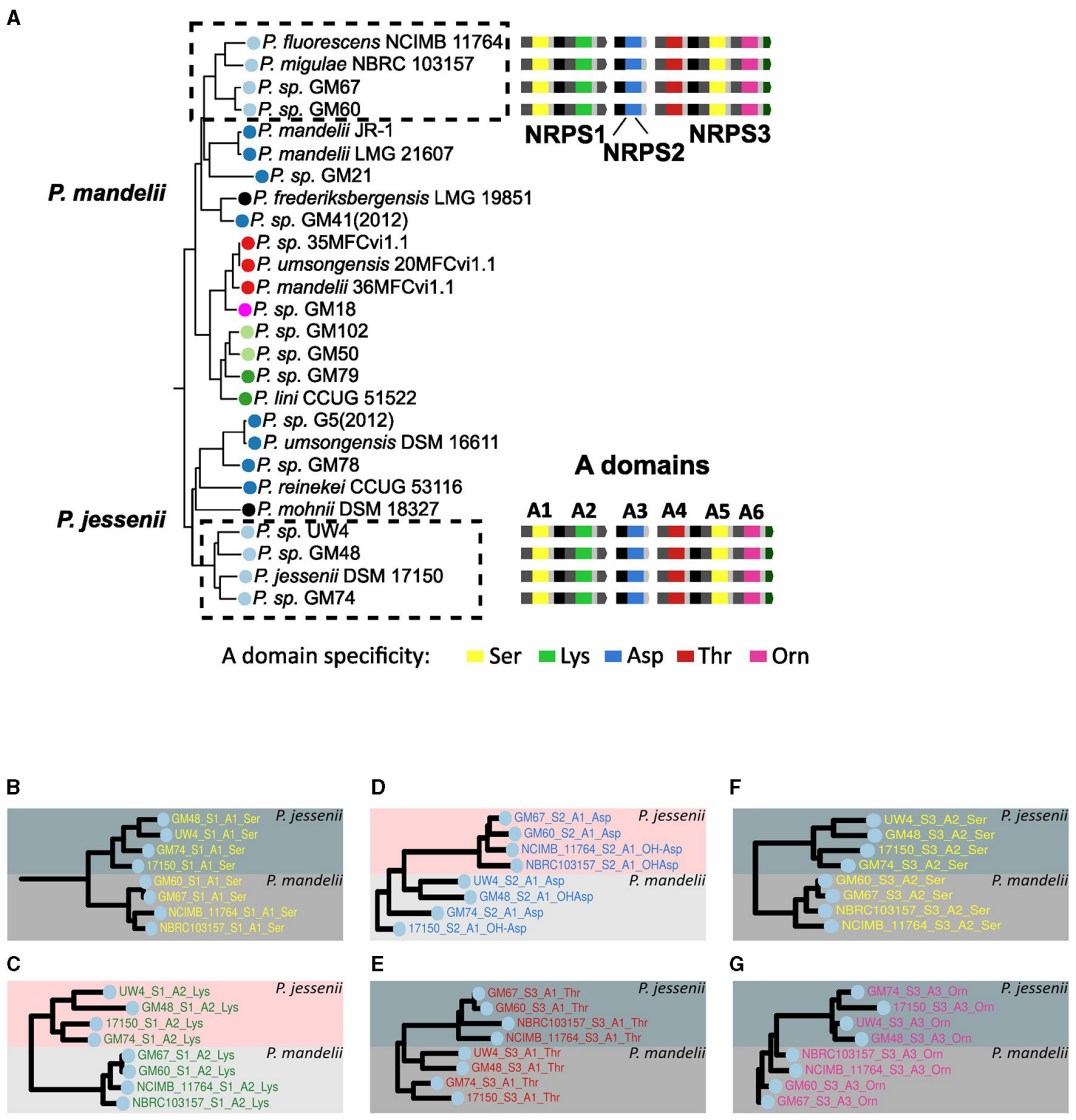
Analysis of the presence of the type I pyoverdine across groups. **(A)** Branches of the phylogenetic tree of species from Figure 1 showing strains bearing the type I pyoverdine, indicated by dashed rectangles. NRPSs and their A domains were assigned numbers to facilitate their identification in this analysis. In a vertical version of the phylogenetic tree of A domains from Figure 4, branches containing A domains from this pyoverdine type were identified. The relevant subtrees of Figure 4 corresponding to A1 **(B)**, A2 **(C)**, A3 **(D)**, A4 **(E)**, A5 **(F)**, and A6 **(G)** domain are amplified.

First, the case of the type I pyoverdine was analyzed. This type features a peptide backbone predicted to be composed of the Ser-Lys-Xxx-Thr-Ser-Orn sequence and is produced by the strains NCIMB 11764, NBRC 103157, GM67 and GM60 constituting a cluster in the *P. mandelii* group and by strains UW4, GM48, DSM 17150 and GM74, from one of the *P. jessenii* group clusters (Figure 5A). To analyze the grouping pattern of the A domains composing this pyoverdine type for each of the strains, the subtrees on which these domains localized were amplified (Figures 5B–G). Within the phylogeny of A domains, the first A domain of the first NRPS (designated the A1 domain) of all species displaying this variant form a single cluster constituted by two subclusters corresponding to the A1 domains of the *P. jesseni* and *P. mandelii* groups (Figure 5B). The same clustering pattern is observed for domains A2–A6 of all the NRPSs for this pyoverdine type (Figures 5C–G). This supports the hypothesis of the full set of NRPSs for the type I pyoverdine being horizontally transferred between an ancestor of a subgroup of the *P. jesseni* group and an ancestor of a subgroup of the *P. mandelii*. Nonetheless, no indication about the direction of this transfer could be inferred from this analysis. Similar analyses were performed with the rest of pyoverdine types distributed in more than one group using the phylogeny of A domains. Evidence for horizontal transfer between groups was found for the NRPSs of types IV and V, as the A domains for these types clustered together in a pyoverdine type-dependent manner (Figure 4).

For the type XVI in the *P. fluorescens* and *P. chlororaphis* group, no clear clustering of the A domains across the groups was observed (Supplementary Figure S2). Notably, the distribution of this type in many branches of the *P. fluorescens* group suggests that this may be an ancestral pyoverdine type produced by this group, which is supported by the ancestral character reconstruction analysis (Figure 3 nodes c and d). However, in this scenario, it is uncertain whether the NRPSs were horizontally transferred across groups or if this represents a situation where one pyoverdine type independently emerged twice.

**Figure 6:**
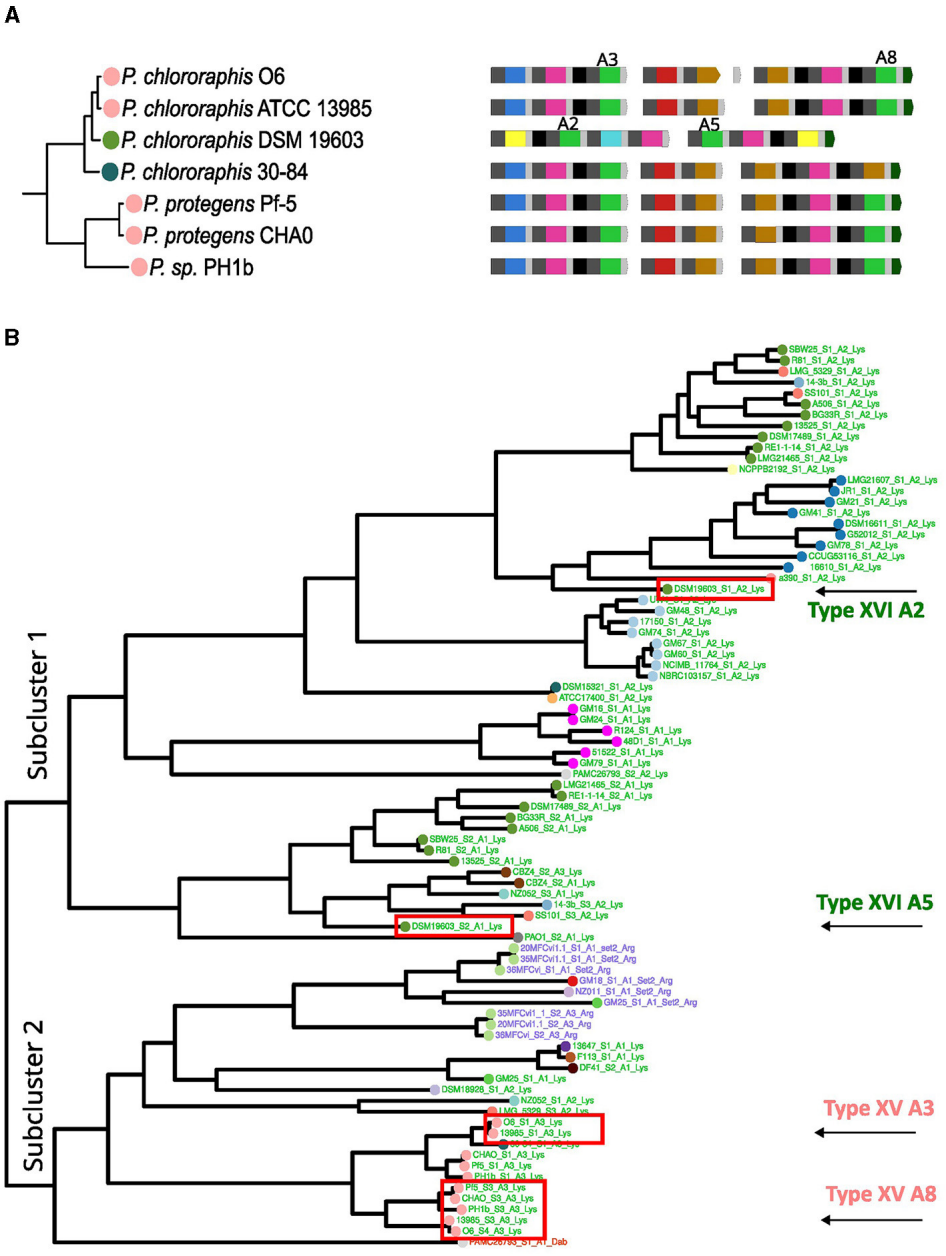
Analysis of the acquisition of type XVI pyoverdine. **(A)** The clusters from the phylogeny of the species corresponding to *Pseudomonas protegens* and *Pseudomonas chlororaphis* with strains bearing type XV and XVI pyoverdine are shown. **(B)** The Lys cluster, from a vertical version of the A domain phylogeny from Figure 4 is shown. The Lys A domain cluster divides into two subclusters. Within these, the A3 and A8 domains for type XIV and A2 and A5 for type XV are identified.

Another change identified was the acquisition of a new set of NRPSs by horizontal transfer in *P. chlororaphis* DSM 19603 within the *P. chlororaphis* group. The three NRPSs carried by the strain *P. chlororaphis* ATCC 13985 likely represent the ancestral set of NRPSs in this cluster, producing the 8 amino acid peptide chain corresponding to the type XV pyoverdine variant (Figure 6A). This type was estimated to be ancestral to the *P. chlororaphis* and *P. protegens* groups. In the DSM 19603 strain, these NRPSs seem to have been replaced by two new NRPSs to produce a seven-amino acid peptide chain corresponding to the type XVI pyoverdine. Type XV and type XVI pyoverdines conserve some A domains with the same substrate. The clustering pattern of two of such domains for each type, the Lys domains (A2 and A5 for type XVI and A3 and A8 for type XV), was investigated. For this, the Lys subtree in the phylogeny of A domains from Figure 4 was analyzed. In general, the cluster for Lys domains splits into two major subclusters. Lys domains for the type XV group together in subcluster 2, while those of the type XVI localize scattered in the subcluster 1 (Figure 6B). This is in accordance with horizontal acquisition of the NRPSs for the type XVI pyoverdine.

### 3.3 Generation of pyoverdine diversity by modification of the original set of NRPSs

**Figure 7:**
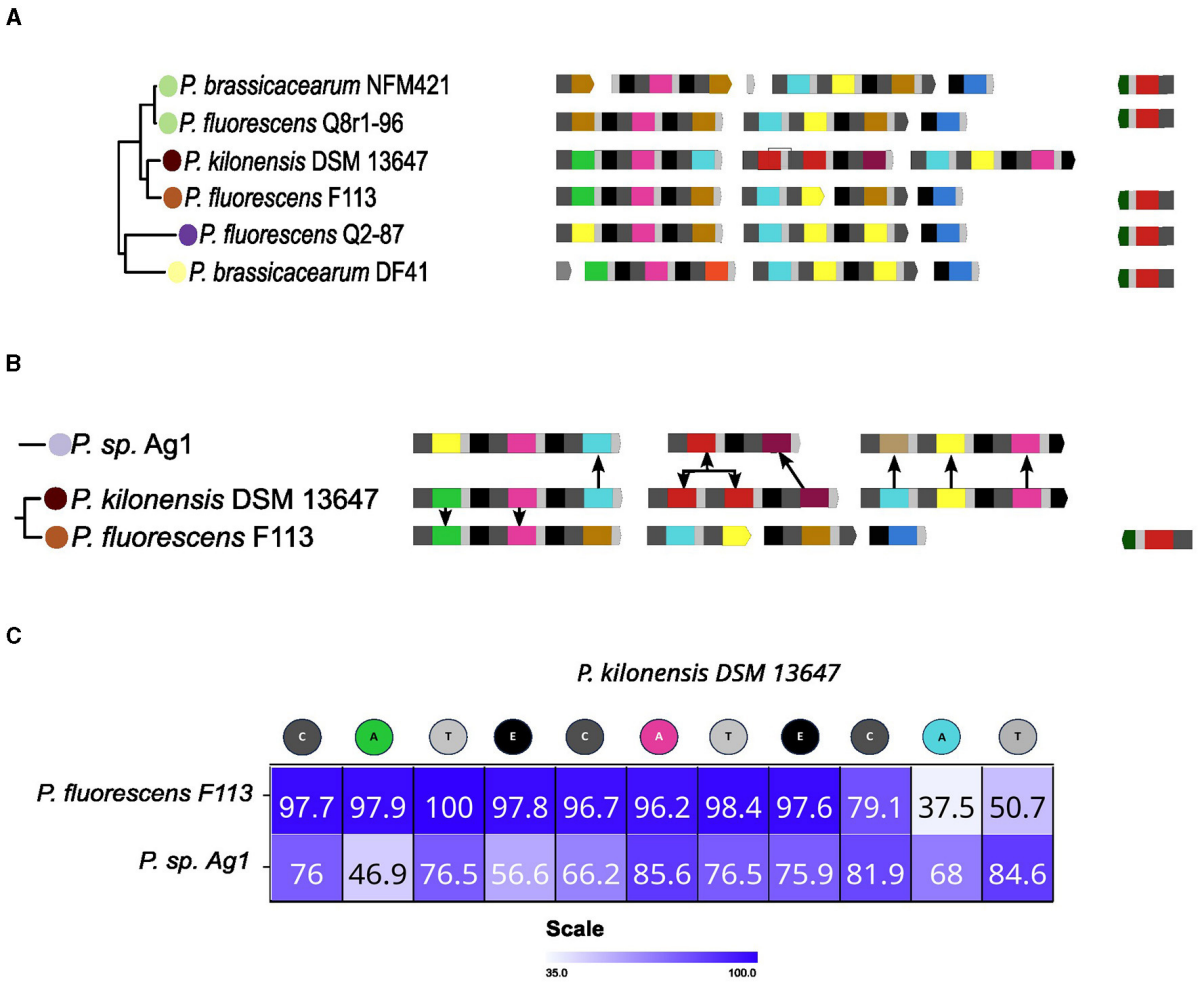
Possible origin of the type XI pyoverdine. **(A)** The *Pseudomonas corrugata* group, which includes *Pseudomonas kilonensis* DSM 13647 on which likely a partial substitution of the NRPRs set occurred, is shown. **(B)** The closest A domains to the domains of the NRPRs for the type XI pyoverdine of DSM 13647 in the A domains phylogenetic tree from Figure 4 are indicated. **(C)** Sequence comparison between the NRPS1 of *P. kilonensis* DSM 13647 and the NRPS1 of *Pseudomonas fluorescen*s F113 and *P. sp*. Ag1. Identity level among domains of the NRPSs is shown as a heat map and indicated by percentage of identity within the heat map squares.

In the cases examined before, it appears that a complete set of NRPSs required for producing the pyoverdine peptide backbone has been horizontally transferred to replace the previous set. However, in the case of *P. kilonensis* DSM 13647, displaying type XI pyoverdine, it appears that a partial substitution of the NRPSs set occurred. For this strain, the ancestral NRPSs set seems to be that conserved in the related *P. fluorescens* F113 (Figure 7A). This set comprises four NRPSs for type XII pyoverdine, with NRPS2 and NRPS3 found fused in adjacent strains. Concerning the composition of the DSM 13647 set, it appears that NRPS1 was partially conserved, while the other NRPSs were lost, and the acquisition of two new NRPSs has taken place. In order to test this, the grouping pattern of the A domains was analyzed. In Figure 7B, the closest A domains in the phylogenetic tree to each A domain of the NRPSs of the *P. kilonensis* DSM 13647 strain are indicated. In agreement with the notion of NRPS1 in *P. kilonensis* DSM 13647 being ancestral, the A1 and A2 domains cluster with the corresponding A domains from *P. fluorescens* F113 and *P. brassicacearum* DF41, which are its neighboring strains in the clade. However, A3, which is the last A domain of NRPS1, as well as A4 to A6 from NRPS2 and NRPS3 were most closely associated with the corresponding A domains of the NRPSs found in *P. sp*. Ag1, from the relatively distant *P. gessardi* group (Figure 7B). This grouping pattern suggests that the first two modules of the NRPS1 of *P. kilonensis* DSM 13647 were maintained, while its C-terminal domain, along with NRPS2 and NRPS3, were likely acquired from a strain related to *P. sp*. Ag1. In accordance, sequence analysis of the NRPS1 indicates that the first two modules share high levels of identity with those of the NRPS1 of *P. fluorescens* F113, while the identity of the domains composing the last module share higher identity with those of NRPS1 of Ag1 (Figure 7C). Notably, the NRPS2 of DSM 13647 has one extra A domain compared to that of Ag1, corresponding to an additional Thr domain (Figure 7B). In the A domain phylogeny, this domain clusters adjacent to its preceding Thr domain in the same NRPS. This suggests the possibility of an intragenic duplication of the Thr domain in this type.

Overall, likely the NRPS1 of DSM 13647 was partially retained while the rest of the NRPSs were substituted by two new enzymes. This analysis indicates that this acquisition event may have been preceded or followed by an A domain duplication. Notably, the cases described so far highlight the role of horizontal transfer as a contributing factor to pyoverdine variation.

**Figure 8:**
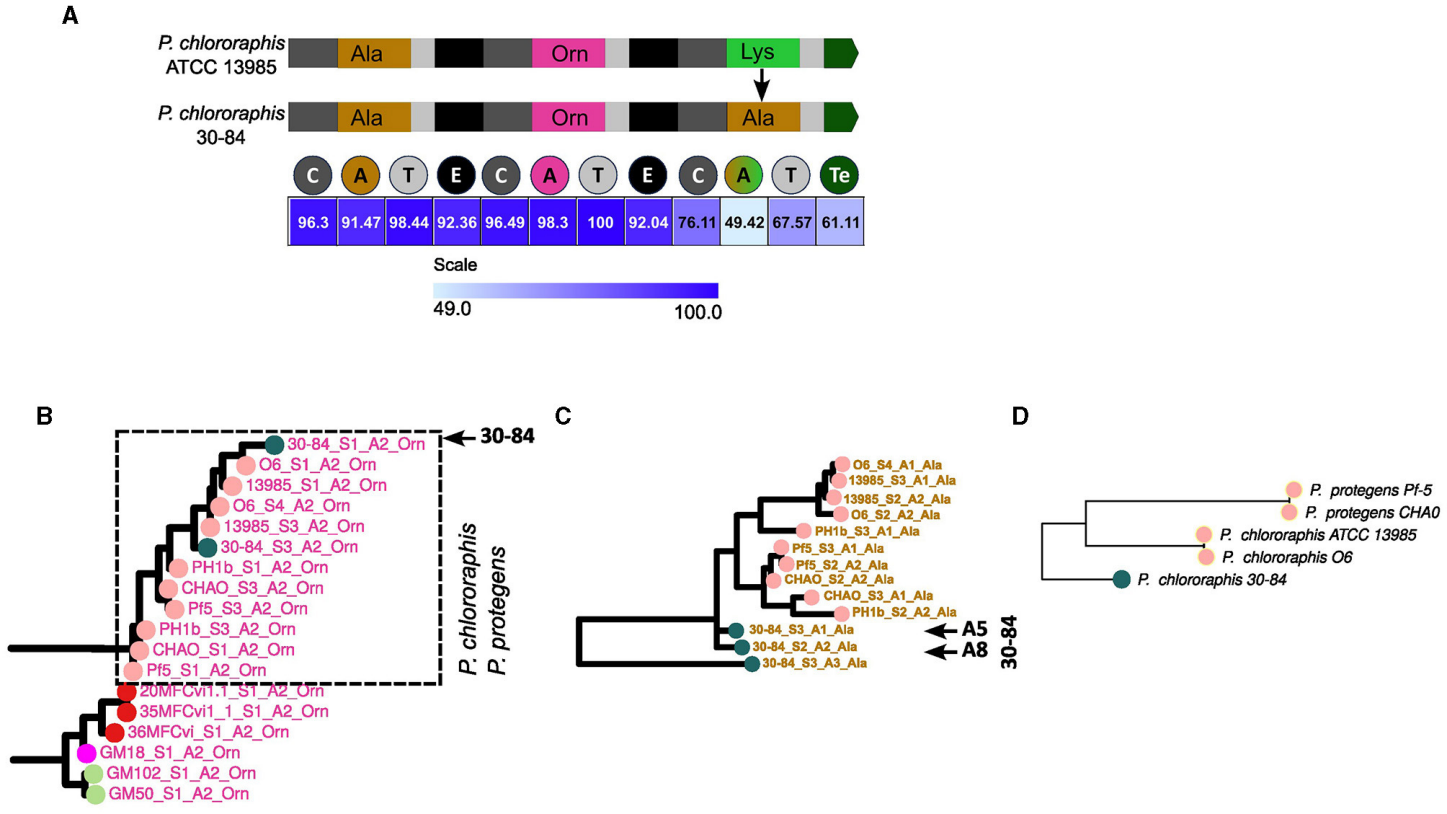
Change of specificity to give rise to type XVII pyoverdine. **(A)** Comparison of NRPS3 of *Pseudomonas chlororaphis* ATCC 13985 (type XV) vs. NRPS3 of *P. chlororaphis* 30–84 (type XVII). The change in specificity of the last A module is indicated. In the bottom, a heat map with the identity levels of each domain between these two proteins is displayed. The subtrees from the A domain phylogeny (Figure 4) including a representative of the conserved domains between the two types (domain A7) **(B)** and the changed domain A8 **(C)** are shown to analyze the clustering pattern. **(D)** Crop of the phylogenetic tree of receptors in Figure 2 showing the clustering pattern of the receptors of strains bearing the type XV and XVII pyoverdines.

A different mechanism of modification identified was the change of specificity of A domains. A clear example of this is the NRPS3 of *P. choloraphis* 30–84. This strain possesses the type XVII pyoverdine. This type is composed of an eight amino acid chain that seems to be a variant of type XV, from which it only differs in the last amino acid (Ala instead of Lys; Figure 6A). As analyzed before, the three NRPSs for the type XIV featured by *P. chlororaphis* ATCC 13985 seem to represent the ancestral set. Thus, the NRPS 3 from *P. choloraphis* 30–84 is a variation of the original NRPS that likely experienced a change of specificity in the third A domain (domain A8 in the whole pathway; Figure 8A). Accordingly, in the phylogeny of A domains, the A domains of the three NRPS producing this variant cluster with their corresponding domains in the NRPSs of the type XIV (for example domain A7 in Figure 8B), except for the A8 domain. Notably, the A8 domain is located within a cluster where its closer relatives are Ala A domains of the NRPSs for this very same pyoverdine type, namely the A6 and A7 domains and their equivalents in neighboring strains (Figure 8C). Furthermore, an alignment of the NRPS3 of *P. choloraphis* 30–84 and the NRPS3 of *P. choloraphis* ATCC 13985, a representative of the strains producing the type XIV, shows a high identity level (>90%) between these two proteins except for the domains comprising the last module and the C terminal Te domain (Figure 8A). In this alignment the identity is particularly low for the last A domain. This strongly suggests that an A domain substitution has occurred in the NRPS3 to give rise to the type XVI variant. Notably this change may have also involved the rest of the last module and the Te domain. In the phylogenetic three of the pyoverdine receptors, the receptors of the type XIV form a cluster composed by two subclusters corresponding to the receptors of strains from *P. chlororaphis* and *P. protegens* groups. The receptor of the type XVI variant associates with this cluster but is the most differentiated protein (Figure 8D). Thus, it seems that the variation of the last amino acid of the ferribactin associates with some degree of adaptive change in the receptor.

**Figure 9:**
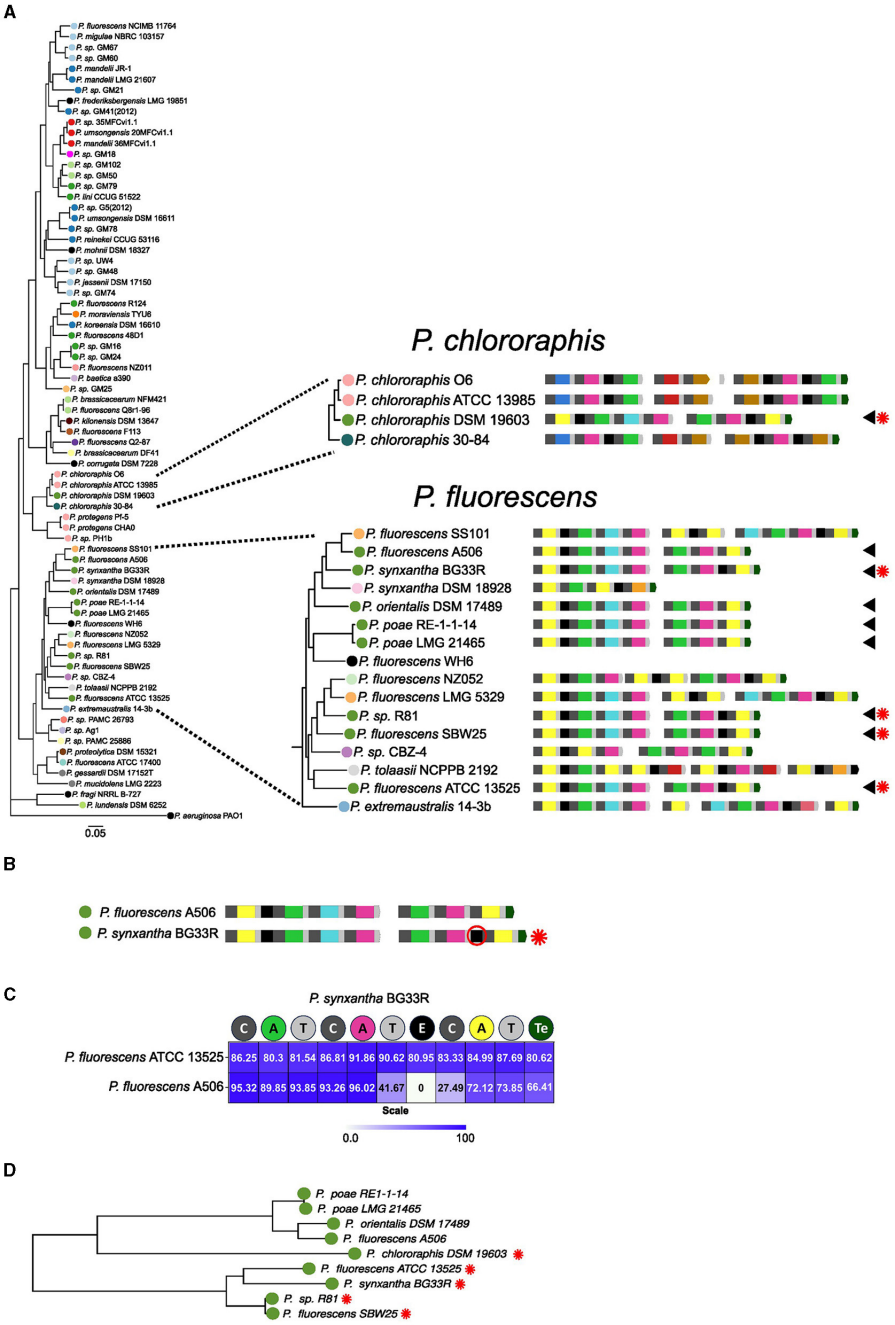
Evidence for gain/loss accessory domain in type XVI pyoverdines. **(A)** Branches of the phylogenetic tree of species corresponding to the *Pseudomonas choloraphis* and *Pseudomonas fluorescens* groups. Strains harboring the type XVI pyoverdine are indicated by arrowheads. The presence of a differential E domain in these strains is indicated by a red asterisk. **(B)** One-on-one comparison of the NRPSs set of *P. fluorescens* A506 and *Pseudomonas syntxantha* BG33R showing the two versions for the type XVI pyoverdine. The differential E domain present in BG33R is highlighted. **(C)** Heat map of percentage of amino acids identity between the NRPS2 of BG33R vs. ATCC 13525 and A506. **(D)** Branch of the phylogenetic tree of receptors including the receptors for the two subtypes of pyoverdine XVI. Receptors for the version including the additional E domain are indicated by asterisks.

This analysis identified two putative versions of the type XVI pyoverdine. As described before, this type is displayed mainly by strains of the *P. fluorescens* group, but also by *P. choloraphis* DSM 19603, from the *P. chlororaphis* group (Figure 9A). The two variants of this type are differentiated by the presence of an E domain in the second module of NRPS2 (Figures 9A, B). Thus, these two versions may differ by the presence of D- or L-Orn in the sixth position of the peptide chain. The NRPS2 of the *P. choloraphis* DSM 19603 conserves this differential E domain. A sequence comparison between NRPS2 of *P. synxantha* BG33R which conserves the E domain, with that of *P. fluorescens* A506, a close relative that lacks the E domain and of *P. fluorescens* ATCC 13525 which also conserves the additional E domain was performed. This evidenced reduced identity for the neighboring T and C domains with those of the protein that lacked the extra accessory domain (Figure 9C). This suggests that the event of E domain gain/loss also involved changes in the surrounding sequences. Independently of the position of the species in the phylogeny, the receptors for these pyoverdines group mainly according to the subtype (Figure 9D). This suggests that these two sets of NRPSs synthesize two distinguishable variants. An exception to this is the receptor of *P. chlororaphis* DSM 19603; however, as it originates from a different group, this receptor may have additional alterations that obscure its pyoverdine-type characteristics. The presence of the E domain in a subset of NRPSs producing the type XV pyoverdine and the clustering of receptors based on the presence/absence of this domain suggests that pyoverdine variation also emerges by the gain/loss of accessory domains in the NRPSs.

## 4 Discussion

In this study, 30 pyoverdine variants were predicted based on the analysis of NRPS genes in the pyoverdine gene cluster among the 74 strains comprising the *P. fluorescens* complex dataset. It is important to clarify that the determination of the pyoverdine types is based on the predictions of three independent methods and although a high accuracy was estimated for the pipeline, experimental determination is lacking for most of the strains included. Given the rapid evolution of this trait, it is very difficult to infer the ancestral pyoverdine of the complex. Furthermore, these analyses can be biased when dealing with characters that are probably under strong selection (Holland et al., 2020). Nonetheless, the ancestral character state reconstruction identified putative original pyoverdines common to the *P. mandelii* and *P*. *jessenii* clades, the *P. chlororaphis* and *P. protegens* clades and an ancestor of most of the strains of the *P. fluorescens* group. These results highlight the fact that despite the high variability, some strains may still conserve mainly unmodified pyoverdines.

In the dataset analyzed, only five strains lack NRPSs for the production of pyoverdine. In all five cases, they have lost both the PvdL and the NRPSs for the peptide backbone. However, two of these strains conserve a pyoverdine receptor that clusters with receptors of strains phylogenetically related. This suggests that these strains may derive from cheaters able to uptake the pyoverdine type they used to synthesize. In the remaining three strains the conservation of pyoverdine receptors for different variants was not investigated, but it is possible that they are able to internalize other types as it has been reported that non-producers tend to express multiple pyoverdine receptors (Butaite et al., 2017). The extreme variability of the Pseudomonads loci for pyoverdine biosynthesis and uptake has been related to the iron-based competition and antagonistic interactions set in the different niches (Smith et al., 2005; Denayer et al., 2007). It has been established that cheaters can use pyoverdines of some but not all producers and may obtain an adaptive advantage under certain conditions. However, pyoverdine variants which are not exploitable are able to inhibit the growth of cheaters by iron deprivation. These interactions are explained by receptor-pyoverdine type compatibility patterns and are dependent on environmental settings (Butaite et al., 2017, 2018; Stilwell et al., 2018). Thus, pyoverdine variation likely has deep implications in different physiological processes, including virulence, leading to the ecological shapes of microbial communities in which *Pseudomonas* participate. Investigating the richness potential and variation strategies of pyoverdines and other siderophores is pivotal to gain insights into the microbiological interactions of this genus.

The modular structure of NRPSs makes them extraordinarily versatile tools for the generation of structural diversity in secondary metabolites. The high diversity of products from NRPS-dependent biosynthesis results from evolutionary processes that take advantage of the autonomy conferred by the modular organization (Brown et al., 2018; Zhang and Kries, 2023). In bacteria, recombination events lie at the core of phenomena involved in NRPSs evolution (Baunach et al., 2021). Evolutionary events known to be involved in the development of novel products include horizontal transfer, gene duplication, bifurcation, point mutations and module and domain duplication and deletion (Götze et al., 2019; Baunach et al., 2021; Booth et al., 2022; Zhang and Kries, 2023). Pyoverdine diversity stands out even among the well documented extreme diversity of molecules and versatility produced by bacterial NRPS. The NRPSs gene clusters for this siderophore display diversification at a level such that identifying evolutionary paths is challenging even when observing strains from the same species. For example, apart from the type IV pyoverdine cluster being a 3.4-kb deletion of the type III, resulting in the elimination of one NRPS module, no other relatedness can be inferred from the reported clusters of *P. aeruginosa* strains. In accordance with previous analysis of NRPS evolution, our study identified several cases of horizontal transfer of NRPS genes. In some instances, the horizontal transfer events rendered strains from different clades putatively producing similar pyoverdines. This is the case of several species of two clades of the groups *P. mandelii* and *P. jessenii*, which share two types of pyoverdine. Notably, this challenges the concept that pyoverdine types are species-specific, largely assumed by the siderotyping procedure (Meyer, 2007; Meyer et al., 2008; Mulet et al., 2008; Ye et al., 2013). Nonetheless, this study did not address the direction of this transfer, nor the role of physiological or ecological traits that may have been involved in such events.

In each instance, the receptors are categorized according to the pyoverdine type. A significant unresolved question pertains to whether the receptor is horizontally transferred alongside the NRPS genes. The receptor gene is located directly downstream of the variable NRPS genes, raising the possibility that this region may also be subject to transfer. Another scenario involves the receptor gene undergoing rapid adaptation to the new pyoverdine produced by the strain. In some cases, the horizontally acquired genes replaced only a subset of the NRPS genes originally present. In the example described, *P. kilonensis* DSM 13647 seems to have conserved the first NRPS and replaced the others with two acquired genes. The analysis of clustering of A domains suggested that the recombination event may have also included the last module of the first gene and a strain of the *P. gessardi* group related to *P*. sp. Ag1. Interestingly, while this alteration results in a distinct type of pyoverdine, unique among the strains examined in this study, the receptor is closely related to the receptor of the *P*. sp. Ag1 variant. This implies that the receptor was also transferred during the process. Analyzing how this receptor evolved to accommodate the pyoverdine produced by the hybrid NRPSs set would be of great interest. In this regard, two strains, *P. proteolytica* DSM 15321 and *P. fluorescens* ATCC 17400, were identified with more than one receptor gene downstream the NRPSs cluster. These species may represent a snapshot of receptor gene multiplication and their study could provide some clues on receptor evolution.

A domain substitution is among the most important events driving NRPS diversification (Baunach et al., 2021; Zhang and Kries, 2023). For example, recombination events changing specificity of A domains led to the production of variants of microcystin (Kurmayer et al., 2005; Baunach et al., 2021), virginiafactins (Götze et al., 2019) and hormaomycin (Höfer et al., 2011). We identified some potential candidates for this event, but the most prominent was the last A domain in type XVII pyoverdine produced by *P. chlororaphis 30–84*. The findings indicate that the alteration might have involved an intra-cluster recombination between this domain and the conserved second A domain of NRPS2 within the same pyoverdine biosynthetic cluster. Understanding the mechanisms behind changes in A domain specificity is crucial for the biotechnological applications of NRPS, as they offer the most straightforward method for producing alternative, non-natural non-ribosomal peptides (Brown et al., 2018). Initial studies suggested that C domains might show specificity for the amino acids charged into a module, implying that both C and A domains need to be replaced when there is a change in module specificity. However, the recombination boundaries leading to these substitutions were not clear (Mootz et al., 2000; Calcott et al., 2014; Bozhüyük et al., 2024). More recent research performed in the PvdD NRPS for pyoverdine biosynthesis in *P. aeruginosa*, showed that novel peptide variants can be obtained by substituting only the A domain without the need to include the cognate C domain, provided that recombination boundaries are finely determined (Calcott et al., 2020). Moreover, when analyzing a large dataset of natural NRPSs, it seems that recombination events that generate diversification involve defined regions within the A domain, namely the A_core_ domain, with a conservation of A domain linkers to adjacent C and T domains (Baunach et al., 2021). Our results reveal that the substitution in the A domain is associated with a noticeable decrease in the identity of the adjacent C and T domains. This indicates that the recombination responsible for the change in the A domain might have also affected these domains. However, a more detailed analysis is needed to define the boundaries that led to the change detected in this study.

Accessory domain gain/loss is also a simple way to generate variation in non-ribosomal peptides. It has been reported that in some strains of the genus *Anabaena* the loss of the sequence for a Methyltransferase (M) domain occurred in the NRPS gene of the microcystin biosynthetic pathway, rendering novel microcystins with distinctive unmethylated dehydroalanine in the produced peptide (Fewer et al., 2008). Our analysis identified a pyoverdine variant that may be alternatively produced with L- or D- ornithine derivatives in the sixth position, owed to the differential conservation of an E domain in the second NRPS of the pathway. This modification probably leads to subtle yet detectable alterations in the final structure of pyoverdine. Despite forming a single clade in the phylogeny, the corresponding receptors tend to cluster based on the presence of the E domain. It has been noticed that integration of E domains into NRPSs is accompanied by specialized T and C domains (Baunach et al., 2021). Accordingly, in this study it was observed a decreased level of identity in the domains flanking the E domain compared to the NRPS version lacking it. This suggests that the gain or loss of the E domain was accompanied by the modification of cognate T and C domains, probably due to stereochemical constraints of the change (Baunach et al., 2021).

Our study found a high variability in NRPSs clusters for the production of the peptide chain of pyoverdine in species of the *P. fluorescens* complex. Nevertheless, the pyoverdine variability was not extensively assessed, as only a subset of known strains belonging to this complex was studied. Hence, many more differences and evolutionary mechanisms are expected to be present in this clade. Additional studies may provide a more comprehensive view of the evolution and details of the processes involved in the variation. Two studies addressing pyoverdine variations and siderophore-based *Pseudomonas* species interactions are in the publishing pipeline at the moment of the preparation of this manuscript (Gu et al., 2023, 2024). These studies extensively assessed the presence of distinctive pyoverdine biosynthesis NRPS and pyoverdine receptors in 1,928 genomes belonging to species of all across the *Pseudomonas* genus and reconstructed the network of pyoverdine utilization in the genus. The authors predicted 188 different pyoverdines and 94 types of receptor groups (Gu et al., 2024). Some of the findings are coincidental with ours. Most notably, the observation that distinct species can produce the same pyoverdine type is corroborated. The pyoverdine peptide chains were predicted to be composed of a core of 13 amino acids plus a few more unidentified which is close to the 12 amino acids core plus unknown substrates we predicted in this study for the *P. fluorescens* complex. Furthermore, high throughput analysis identified recombination driving NRPS domain and subdomain substitutions, indels and replacements as the main general mechanisms for pyoverdine evolution with a role of horizontal transfer in this mechanism (Gu et al., 2023). Our study focused on the *P. fluorescens* complex and took advantage of phylogenetic-based and ancestral state character reconstruction analysis of evolutionary history of pyoverdine. This allowed the identification of the two main mechanisms of pyoverdine variation and specific events of NRPSs clusters modification, including cases of horizontal transfer confirmed by the phylogenetic analysis. Importantly, some insights found in the present study could be further studied applying the co-evolutionary based approaches developed by Gu et al. For example, the undergoing evolution of pyoverdine receptors in the strains with recent receptor duplications pinpointed in this work could be studied by assessing the characteristics of the receptor sequence features defined by Gu et al. (2023).

The accumulation of knowledge on the evolution of non ribosomal peptides processes is needed for the rational design of NRPRs by synthetic biology for novel products (Matsukawa et al., 2023). Perhaps more importantly, the mechanism involved in the variation of pyoverdine/pyoverdine receptors will provide important clues about the physiological and evolutionary rules governing complex microbiological communities.

## Data Availability

The original contributions presented in the study are included in the article/Supplementary material, further inquiries can be directed to the corresponding author.
