# Supplementary material for: Total substitution and partial modification of the set of non-ribosomal peptide synthetases clusters lead to pyoverdine diversity in the Pseudomonas fluorescens complex
Source: Front Microbiol. 2024 Aug 19;15:1421749. doi: 10.3389/fmicb.2024.1421749 (PMC11366639; doi:10.3389/fmicb.2024.1421749)
Supplement: Supplementary file 1 [file Data_Sheet_1.PDF]

Pyoverdine type	Sequence of peptidic chain	Strains
**I**	Ser-Lys-Asp-Thr-Ser-Orn	*P. fluorescens* NCIMB 11764, *P. migulae* NBRC 103157, *P. sp*. GM67, *P. sp*. GM60, *P. sp*. UW4, *P. sp*. GM48, *P. jessenii* DSM 17150, *P. sp*. GM74
**II**	Ala-Lys-Thr-Ser-X-Orn	*P. mandelii* JR-1, *P. mandelii* LMG 21607, *P. sp.* GM21, *P. sp.* GM41(2012), *P. sp.* G5(2012), *P. umsongensis* DSM 16611, *P. sp.* GM78, *P. reinekei* CCUG 53116, *P. koreensis* DSM 16610
**III**	Ala-Orn-Ala-Gly-Ser-Arg-Asp-Arg	*P. sp.* 35MFCvi1.1, *P. umsongensis* 20MFCvi1.1, *P. mandelii* 36MFCvi1.1
**IV**	Ala-Orn-Ala-Gly-Ser-Ala-Asp-Arg	*P. sp.* GM18
**V**	Ala-Orn-Ala-Gly-Ser-Ala-Asp-Thr	*P. sp.* GM102, *P. sp*. GM50, *P. brassicacearum* NFM421, *P. fluorescens* Q8r1-96,
**VI**	Lys-Asp-Gly-Thr-Ala-Orn	*P. sp.* GM79, *P. lini* CCUG 51522, *P. fluorescens* R124, *P. fluorescens* 48D1, *P. sp.* GM16, *P. sp.* GM24
**VII**	Ser-Orn-Ala-Gly-Thr-Ala-Orn	*P. moraviensis* TYU6
**VIII**	Ser-Orn-Ala-Gly-Thr-Ala-Asp-Arg	*P. fluorescens* NZ011
**IX**	Ser-Lys-Ser-Thr-Ala-Orn-Orn	*P. baetica* a390
**X**	Lys-Orn-Ala-Gly-Thr-Ala-Asp-Arg	*P. sp.* GM25
**XI**	Lys-Orn-Gly-Thr-Thr-Gln-Gly-Ser-Orn	*P. kilonensis* DSM 13647
**XII**	Lys-Orn-Ala-Gly-Ser-Ala-Asp-Thr	*P. fluorescens* F113
**XIII**	Ser-Orn-Ala-Gly-Ser-Ser-Asp-Thr	*P. fluorescens* Q2-87
**XIV**	Lys-Orn-X-Gly-Ser-Ser-Asp-Thr	*P. brassicacearum* DF41
**XV**	Asp-Orn-Lys-Thr-Ala-Ala-Orn-Lys	*P. chlororaphis* O6, *P. chlororaphis* ATCC 13985, *P. protegens* Pf-5, *P. protegens* CHA0, *P. sp.* PH1b
**XVI**	Ser-Lys-Gly-Orn-Lys-Orn-Ser	*P. chlororaphis* DSM 19603, *P. fluorescens* A506, *P. synxantha* BG33R, *P. orientalis* DSM 17489, *P. poae* RE-1-1-14, *P. poae* LMG 21465, *P. sp.* R81, *P. fluorescens* SBW25, *P. fluorescens* ATCC 13525
**XVII**	Asp-Orn-Lys-Thr-Ala-Ala-Orn-Ala	*P. chlororaphis* 30-84
**XVIII**	Ser-Lys-Gly-Orn-Ser-Ser-Gly-Lys-Orn-Ser	*P. fluorescens* SS101, *P. fluorescens* LMG 5329
**XIX**	Ser-Lys-Ser-X	*P. synxantha* DSM 18928
**XX**	Ser-Lys-Orn-Ser-Ser-Lys-Orn-Ser	*P. fluorescens* NZ052
**XXI**	Ser-Ser-Orn-Lys-Orn-Lys-Ser	*P. sp.* CBZ-4
**XXII**	Ser-Lys-Ser-Ser-Thr-Ser-Orn-Thr-Ser-X	*P. tolaasii* NCPPB 2192
**XXIII**	Ser-Lys-Gly-Orn-Ser-Gly-Lys-Orn-Glu-Ser	*P. extremaustralis* 14-3b
**XXIV**	Dab-Orn-Lys-Asp-Lys-Ala	*P. sp.* PAMC 26793
**XXV**	Ser-Orn-Gly-Thr-Gln-Phe-Ser-Orn	*P. sp.* Ag1
**XXVI**	Ser-Dab-Asp-Ala	*P. sp.* PAMC 25886
**XXVII**	Ala-Lys-Gly-Thr-Asp-Orn-Ala-Ala-Ala-Ala-Ser-Gln	*P. proteolytica* DSM 15321
**XXVIII**	Ala-Lys-Gly-Gly-Asp-X-Ala-Ser-X-Gln	*P. fluorescens* ATCC 17400
**XXIX**	Ser-Glu-Ala-Ser-Asp-Gly-Gly-Ala-Gly-Orn	P. gessardii DSM 17152T, *P. mucidolens* LMG 2223
**XXX**	Ser-Asp-Thr-Ser-Orn-Orn	*P. lundensis* DSM 6252

Table S1. Pyoverdine types and predicted sequences for the strains used in this study.

Table S2. Comparison of the predicted amino acid sequences in strains with reported pyoverdine sequence. Amino acids not accurately predicted are indicated in bold.

Strain	Predicted sequence	Reported sequence	Reference
P. protegens Pf-5	Asp-Orn-Lys-Thr-Ala-Ala-Orn-Lys	Asp-FOHOrn-Lys-(Thr-Ala-Ala-FOHOrn-Lys)	Hartney, SL. 2013
P. protegens CHA0	Asp-Orn-Lys-Thr-Ala-Ala-Orn-Lys	Asp-FOHOrn-Lys-(Thr-Ala-Ala-FOHOrn-Lys)	Wong-Lun-Sang S. 1996. Tetrahedron Lett.
P. koreensis DSM 16610	Ala-Lys-Thr-Ser-**Xxx**-Orn	Ala-Lys-Thr-Ser-AOHOrn-cOHOrn	Matthijs, S. 2016. Biometals
P. aeruginosa PAO1	Ser-Arg-Ser-Orn-Lys-Orn-Thr-Thr	Ser-Arg-Ser-FOHOrn-(Lys-FOHOrn-Thr-Thr)	Demange, P. 1990. Biometals
P. fluorescens ATCC 13525	Ser-Lys-Gly-Orn-Lys-Orn-Ser	Ser-Lys-Gly-FOHOrn-(Lys-FOHOrn-Ser)	Linget, C., 1992. Tetrahedr Lett
P. fluorescens SBW25	Ser-Lys-Gly-Orn-Lys-Orn-Ser	Ser-Lys-Gly-FOHOrn-(Lys-FOHOrn-Ser)	Moon, CD, 2008. BMC Micro
P. tolaasii NCPPB 2192	Ser-Lys-Ser-Ser-Thr-Ser-Orn-Thr-Ser-**Xxx**	Ser-Lys-Ser-Ser-Thr-Ser-OHOrn-Thr-Ser-OHOrn	Demange, P. 1990. Biochem
P. brassicacearum NFM421	**Ala**-Orn-Ala-Gly-Ser-Ala-Asp-Thr	Ser-AcOHOrn-Ala-Gly-(Ser-Ala-OHAsp-Thr	Matthijs, S. 2016. Biometals

Figure S1

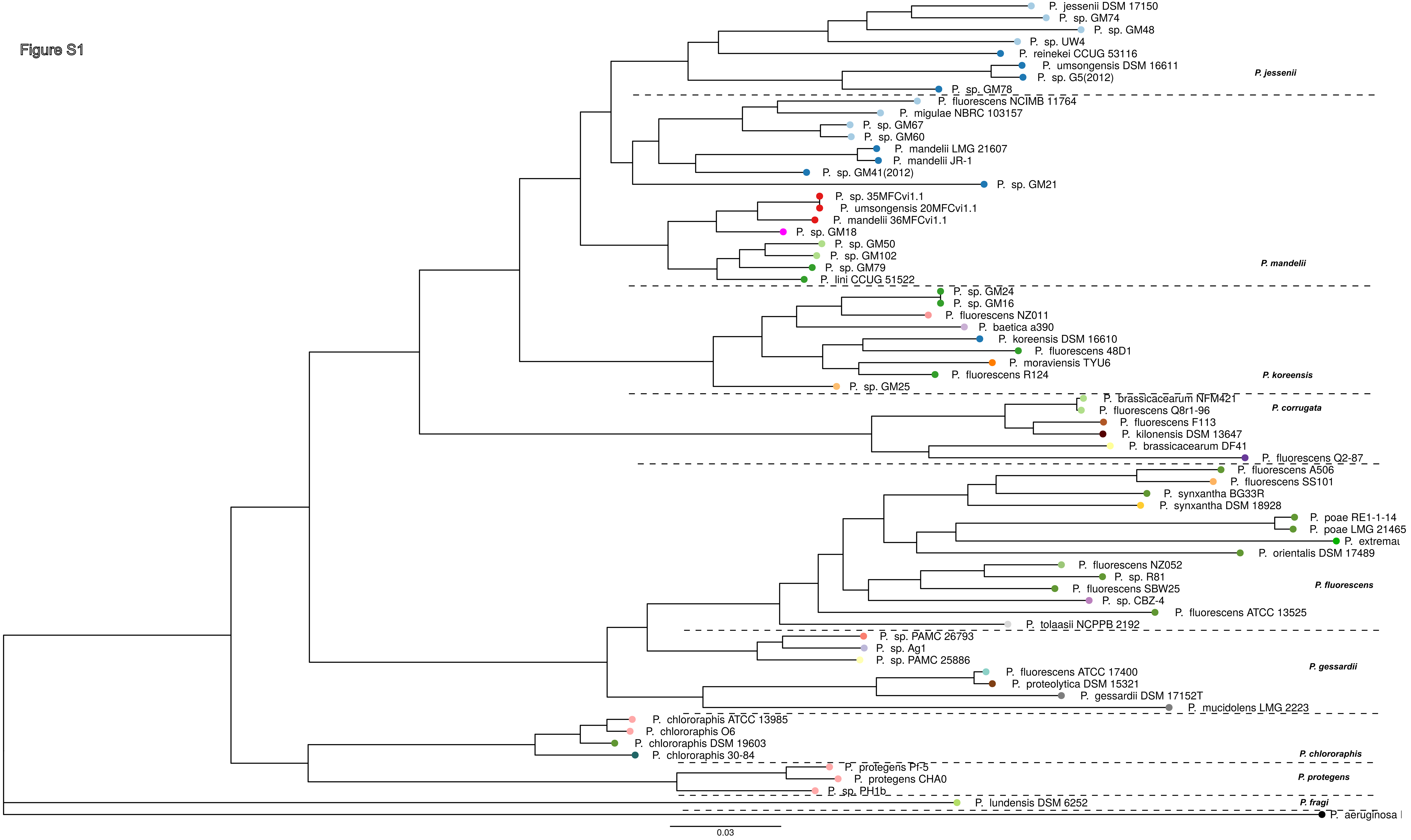
P. jessenii DSM 17150
P. sp. GM74
P. sp. GM48
P. sp. UW4
P. reinekei CCUG 53116
P. umsongensis DSM 16611
P. sp. G5(2012)
P. sp. GM78
P. jessenii
P. fluorescens NCIMB 11764
P. migulae NBRC 103157
P. sp. GM67
P. sp. GM60
P. mandelii LMG 21607
P. mandelii JR-1
P. sp. GM41(2012)
P. sp. GM21
P. sp. 35MFCvi1.1
P. umsongensis 20MFCvi1.1
P. mandelii 36MFCvi1.1
P. sp. GM18
P. sp. GM50
P. sp. GM102
P. sp. GM79
P. lini CCUG 51522
P. mandelii
P. sp. GM24
P. sp. GM16
P. fluorescens NZ011
P. baetica a390
P. koreensis DSM 16610
P. fluorescens 48D1
P. moraviensis TYU6
P. fluorescens R124
P. sp. GM25
P. koreensis
P. brassicacearum NFM421
P. fluorescens Q8r1-96
P. corrugata
P. fluorescens F113
P. kilonensis DSM 13647
P. brassicacearum DF41
P. fluorescens Q2-87
P. fluorescens A506
P. fluorescens SS101
P. synxantha BG33R
P. synxantha DSM 18928
P. poae RE1-1-14
P. poae LMG 21465
P. extremau
P. orientalis DSM 17489
P. fluorescens NZ052
P. sp. R81
P. fluorescens SBW25
P. sp. CBZ-4
P. fluorescens ATCC 13525
P. fluorescens
P. tolaasii NCPPB 2192
P. sp. PAMC 26793
P. sp. Ag1
P. sp. PAMC 25886
P. fluorescens ATCC 17400
P. proteolytica DSM 15321
P. gessardii DSM 17152T
P. mucidolens LMG 2223
P. gessardii
P. chlororaphis ATCC 13985
P. chlororaphis O6
P. chlororaphis DSM 19603
P. chlororaphis 30-84
P. chlororaphis
P. protegens Pf-5
P. protegens CHA0
P. protegens
P. sp. PH1b
P. lundensis DSM 6252
P. fragi
P. aeruginosa l
0.03

Figure S1. Phylogenetic tree based on the alignment of 70 PvdL sequences. The circle colors indicate the pyoverdine synthesized by the corresponding strain as in Figure 1. The previously defined groups within the complex are outlined by dashed lines.

Figure S2

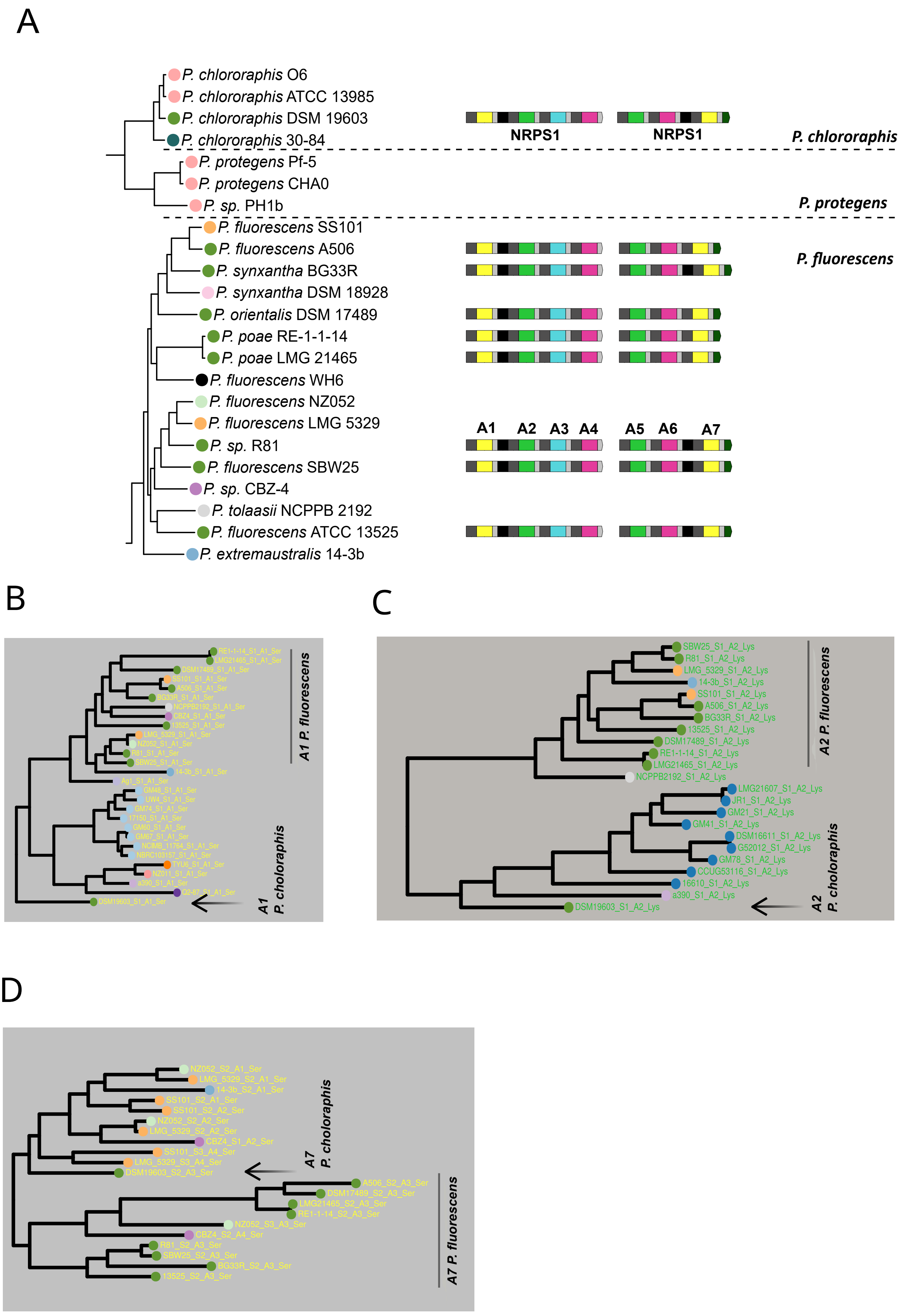
A
P. chlororaphis O6
P. chlororaphis ATCC 13985
P. chlororaphis DSM 19603
P. chlororaphis 30-84
P. protegens Pf-5
P. protegens CHA0
P. sp. PH1b
P. fluorescens SS101
P. fluorescens A506
P. synxantha BG33R
P. synxantha DSM 18928
P. orientalis DSM 17489
P. poae RE-1-1-14
P. poae LMG 21465
P. fluorescens WH6
P. fluorescens NZ052
P. fluorescens LMG 5329
P. sp. R81
P. fluorescens SBW25
P. sp. CBZ-4
P. tolaasii NCPPB 2192
P. fluorescens ATCC 13525
P. extremaustralis 14-3b
NRPS1
NRPS1
P. chlororaphis
P. protegens
P. fluorescens
A1
A2
A3
A4
A5
A6
A7
B
A1 P. fluorescens
A1
P. choloraphis
C
SBW25_S1_A2_Lys
R81_S1_A2_Lys
LMG_5329_S1_A2_Lys
14-3b_S1_A2_Lys
SS101_S1_A2_Lys
A506_S1_A2_Lys
BG33R_S1_A2_Lys
13525_S1_A2_Lys
DSM17489_S1_A2_Lys
RE1-1-14_S1_A2_Lys
LMG21465_S1_A2_Lys
NCPPB2192_S1_A2_Lys
LMG21607_S1_A2_Lys
JR1_S1_A2_Lys
GM21_S1_A2_Lys
GM41_S1_A2_Lys
DSM16611_S1_A2_Lys
G52012_S1_A2_Lys
GM78_S1_A2_Lys
CCUG53116_S1_A2_Lys
16610_S1_A2_Lys
a390_S1_A2_Lys
DSM19603_S1_A2_Lys
A2 P. fluorescens
A2
P. choloraphis
D
NZ052_S2_A1_Ser
LMG_5329_S2_A1_Ser
14-3b_S2_A1_Ser
SS101_S2_A1_Ser
SS101_S2_A2_Ser
NZ052_S2_A2_Ser
LMG_5329_S2_A2_Ser
CBZ4_S1_A2_Ser
SS101_S3_A4_Ser
LMG_5329_S3_A4_Ser
DSM19603_S2_A3_Ser
A506_S2_A3_Ser
DSM17489_S2_A3_Ser
LMG21465_S2_A3_Ser
RE1-1-14_S2_A3_Ser
NZ052_S3_A3_Ser
CBZ4_S2_A4_Ser
R81_S2_A3_Ser
SBW25_S2_A3_Ser
BG33R_S2_A3_Ser
13525_S2_A3_Ser
A7
P. choloraphis
A7 P. fluorescens

Figure S2. Analysis of the distribution of Type XVI pyoverdine. A. Branches of the phylogenetic tree of species from Fig. 1 showing strains bearing the type XVI pyoverdine, with the cognate NRPS shown. NRPSs and their A domains were assigned numbers to facilitate their identification in this analysis. In a vertical version of the phylogenetic tree of A domains from Fig. 4, branches containing domains from this pyoverdine type were identified and amplified here. Equivalent A domains from the *P. fluorescens* and *P. choloraphis* subgroups localize in different clusters. Examples A1 (**B**), A2 (**C**) and A7 (**D**) are depicted.
